# Effectiveness of regionally-specific immunotherapy for the management of canine atopic dermatitis

**DOI:** 10.1186/s12917-016-0917-z

**Published:** 2017-01-05

**Authors:** Jon D. Plant, Moni B. Neradilek

**Affiliations:** 1 SkinVet Clinic, 15800 Upper Boones Ferry Road, Suite 120, Lake Oswego, 97035 OR USA; 2 The Mountain-Whisper-Light Statistics, 1827 23rd Avenue East, Seattle, 98112 WA USA

**Keywords:** Dog, Atopic dermatitis, Regionally-specific immunotherapy, RESPIT, Allergen, Immunotherapy, Pruritus

## Abstract

**Background:**

Canine atopic dermatitis is a common pruritic skin disease often treated with allergen immunotherapy (AIT). AIT in dogs traditionally begins with attempting to identify clinically relevant environmental allergens. Current allergen testing methodologies and immunotherapy techniques in dogs are not standardized. Immunotherapy with a mixture of allergenic extracts selected based on regional aerobiology rather than intradermal tests or serum IgE assays has been described. The objective of this study was to evaluate the effectiveness of regionally-specific immunotherapy in dogs with atopic dermatitis. The medical records of a veterinary dermatology referral clinic were searched for dogs with atopic dermatitis that began regionally-specific subcutaneous immunotherapy from June, 2010 to May, 2013. An overall assessment of treatment effectiveness (excellent, good, fair, or poor) was assigned based upon changes in pruritus severity, lesion severity, and the reduction in concurrent medication(s) during a follow-up period of at least 270 days. Baseline characteristics that might predict treatment success were analyzed with the Spearman’s correlation and the Kruskal-Wallis tests.

**Results:**

Of the 286 dogs that began regionally-specific immunotherapy (RESPIT) during a 3 year period, 103 met the inclusion criteria. The overall response to RESPIT was classified as excellent in 19%, good in 38%, fair in 25%, and poor in 18% of dogs. The response classification correlated significantly with a reduction in pruritus severity (*r* = 0.72, *p* < 0.001) and lesion severity (*r* = 0.54, *p* < 0.001), but not with the dogs’ baseline characteristics. Adverse reactions were reported in 7/286 (2.4%) of treated dogs.

**Conclusions:**

Under the conditions of this study, RESPIT was safe and effective for the treatment of atopic dermatitis in dogs.

## Background

Canine atopic dermatitis (AD) is a common inflammatory and pruritic skin disease that is frequently associated with sensitization to environmental allergens [1]. Affected dogs often exhibit pruritus of the face, pinnae, feet, axillae, and inguinal region [2]. Secondary otitis externa, staphylococcal pyoderma and *Malassezia* dermatitis frequently develop in atopic dogs.

Canine AD often requires long-term management and therapy [1]. There is substantial evidence to support the use of glucocorticoids, cyclosporine, oclacitinib, and allergen immunotherapy (AIT) for canine AD [3]. With AIT, dogs are given allergenic extracts in order to minimize flares upon subsequent natural exposure [4]. The mechanism of action of AIT is not well defined in dogs, but may include the production of blocking IgG antibodies, a shift in the cytokine balance from a predominantly T-helper (Th) 2 to a Th1 cell profile, and a regulatory T-cell response [5, 6]. Therapeutic allergens are identified through a combination of aerobiology, intradermal test (IDT) findings, serum allergen-specific IgE assays (SIA), and clinical history [2]. Allergenic extracts are administered either by subcutaneous injection or via application to the oral mucosa [3].

Allergen immunotherapy prescriptions are customized for each dog. An optimal allergenic extract mixture would contain only allergens that elicit clinical signs upon natural exposure. However, customizing an allergenic extract involves multiple subjective variables. Veterinarians choose which allergens to test for, whether to test with IDT or SIA, which laboratory’s SIA to use, how to interpret borderline reactions, which positive reactions are deemed clinically relevant, what dose of each allergen to include in the ASIT prescription, and by what schedule and route it will be administered. These variables are not trivial. Within a geographic region, the allergens veterinary dermatologists evaluate with IDT vary substantially [7], as do the allergens assayed by different laboratories offering SIA [8]. The agreement between IDT and SIA findings is often poor [2]. False positive and false negative results occur with both testing methods [1]. Recently, Plant et al. found poor agreement between four SIAs, indicating that the choice of laboratories is likely to influence treatment recommendations [9]. Once allergens are selected for inclusion, the optimal dose of each is unknown in veterinary medicine. Allergen immunotherapy may, therefore, be considered a heterogeneous therapy.

Although subject to the variables described above, AIT has been found to be effective for the management of canine AD in one placebo-controlled and multiple retrospective studies [5]. Results from these studies are difficult to compare directly because they report different outcome measures, but those that defined effectiveness as a greater than 50% reduction in pruritus and lesion severity found AIT to be effective in 52–77% of dogs [5]. In most of these studies, the response to AIT was independent of the testing method, the age of onset of AD, the age at which AIT was begun, and the duration of disease prior to AIT. Mixed findings have been reported concerning the correlation of treatment success with breed, gender, and the seasonality of signs [5].

An alternative to AIT is RESPIT, allergenic extract mixtures that are formulated based on a dog’s geographic location rather than individual allergy test findings. Reports on the use of non-specific AIT mixtures in dogs or humans are limited [10–12]. The aim of this study was to evaluate the effectiveness of subcutaneous RESPIT for the treatment of atopic dermatitis in 103 dogs that began therapy during a 3-year period at a veterinary dermatology clinic in the northwestern United States.

## Methods

The electronic medical records (Vetport, Vetport, LLC, Milford, OH, USA) of a veterinary dermatology clinic were searched for dogs with AD for which RESPIT (RESPIT® Injectable Region 1, Respit, LLC, Lake Oswego, OR, USA) was prescribed on the same date as an initial examination between June 1, 2010 and May 31, 2013. The diagnosis of AD was made based on identifying characteristic clinical features and ruling out alternative diagnoses [13].

The following history and examination findings from the initial encounter on day 0 (D0) were exported to a spreadsheet: patient identification, date of birth, gender, breed, weight, encounter date, pruritus visual analogue scale (PVAS) [14], seasonality of signs, current medications, and lesion severity. Lesion severity was recorded with an ad hoc canine lesion severity index (LSI) with a range from 0 to 1000 (the product of lesion severity graded from 0 to 10 and estimated percent body area affected). Dogs without a D0 PVAS entry were excluded from analysis. The records of the remaining dogs were reviewed to identify those that returned for an examination after receiving RESPIT for at least 270 days. Nine to twelve months is often the duration of therapy recommended to evaluate the response to AIT in dogs [15, 16]. The date of the first examination following 270 days of RESPIT therapy (designated D270+) was recorded and the following data were further extracted from the medical records: D270+ PVAS, D270+ LSI, D270+ concurrent medication(s), and adverse reactions suspected by the pet owner. The last date that RESPIT was dispensed before July 15, 2015 was also recorded.

On the basis of the changes in the dogs’ PVAS, LSI, and those concomitant systemic medications with substantial evidence of efficacy (glucocorticoids, cyclosporine, or oclacitinib) between D0 and D270+, an overall assessment score was assigned by the primary investigator as follows: 1 (poor) = no clinical change or a deterioration, 2 (fair) = improvement, but concurrent medications could not be substantially decreased, 3 (good) = greater than 50% improvement in clinical signs and reduction in medications, 4 (excellent) = complete remission without concurrent medications [17]. The percentages of dogs with D270+ PVAS in the normal (<2.0) and mild (2.0–3.5) ranges were determined.

All statistical analyses were carried out in the statistical software R: A Language and Environment for Statistical Computing (R Foundation for Statistical Computing, Vienna, Austria) version 3.1.3. Continuous and categorical characteristics were analyzed with Spearman’s correlation and the Kruskal-Wallis test, respectively. A *P*-value of <0.05 was considered statistically significant. Trends in bivariate relationships were highlighted by local regression trend lines [18].

## Results

During a 3 year period, 286 dogs with AD began RESPIT on the day of an initial examination during which the pruritus severity was recorded. Of these dogs,103 (36%) returned for an examination after 270 days while still receiving RESPIT, thereby meeting the inclusion criteria. Most commonly, dogs were excluded due to poor compliance. Eighty-five dogs received only the initial prescription of RESPIT, an insufficient volume to last 270 days. Three of these dogs transitioned to an oromucosal formulation of RESPIT. Fifty-five dogs had their prescriptions renewed for the last time before day 200 and likely did not receive a volume sufficient to reach D270+. These 55 dogs were not examined after D270 while receiving subcutaneous RESPIT. Two of these dogs also transitioned to the oromucosal formulation of RESPIT. D270+ examinations while receiving RESPIT were not performed for 39 dogs that did have RESPIT prescriptions renewed after day 200. Four additional dogs had lengthy (7 month to 4 year) lapses in therapy before the D270+ examinations. The D0 baseline characteristics (age, weight, gender, seasonality of signs, PVAS, and LSI) of the included (*n* = 103) and excluded (*n* = 183) dogs were not significantly different (*p* > 0.05).

**Fig. 1:**
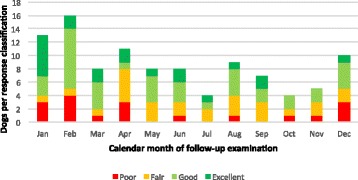
Effectiveness of regionally-specific immunotherapy by calendar month of follow-up examination in 103 dogs with atopic dermatitis

**Fig. 2:**
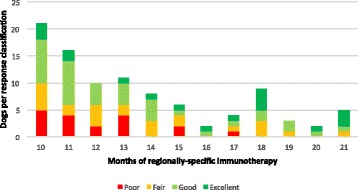
Effectiveness of regionally-specific immunotherapy by duration of treatment in 97 atopic dogs. Dogs with follow-up examinations beyond 21 months (2 classified as excellent, 2 as good, and 2 as fair) are not depicted

The overall response to RESPIT was scored as excellent in 19%, good in 38%, fair in 25%, and poor in 18% of dogs. The percentages of dogs with normal or mild pruritus at D270+ were 20 and 25%, respectively. The mean duration of therapy evaluated was 424 days (median 365, range 273–1735 days). D0 age (*r* = 0.06, *p* = 0.5), weight (*r* = −0.03, *p* = 0.7), gender (*p* = 0.2), pruritus severity (*r* = 0.09, *p* = 0.4), lesion severity (*r* = 0.10, *p* = 0.3), seasonal history (*p* = 0.2), and the calendar month of the D270+ examination (*p* = 0.8, Fig. 1) did not correlate significantly with the response classification. The number of dogs assigned to each response classification per 30-day period following D270+ is depicted in Fig. 2. Ninety percent of dogs scored as excellent and 33% of dogs scored as good were not receiving anti-pruritic medications at the time of the D270+ examination (Table 1). One dog scored as excellent was receiving occasional oral diphenhydramine and a second infrequent topical fluocinonide cream.

**Table 1:** Number of dogs per response classification receiving concomitant anti-pruritic medications with RESPIT at D270+

Concomitant anti-pruritic medication	Poor *n* = 18	Fair *n* = 26	Good *n* = 39	Excellent *n* = 20
None	4 (22%)	6 (23%)	13 (33%)	18 (90%)
Oral glucocorticoid	7 (39%)	10 (38%)	17 (44%)	0 (0%)
Cyclosporine	4 (22%)	7 (27%)	7 (18%)	0 (0%)
Oclacitinib	0 (0%)	0 (0%)	2 (5%)	0 (0%)
Antihistamine	3 (17%)	5 (19%)	1 (3%)	1 (5%)
Topical glucocorticoid, including otic	1 (6%)	1 (4%)	0 (0%)	1 (5%)

5/103 dogs were receiving two classes of medications

**Fig. 3:**
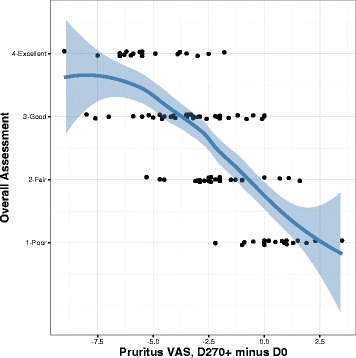
Effectiveness of regionally-specific immunotherapy in dogs versus change in pruritus severity. The change in the PVAS equals the PVAS at D270+ minus the PVAS at D0. The line is a locally weighted scatterplot smoother with the shaded area showing the 95% confidence interval around the point-wise mean

**Fig. 4:**
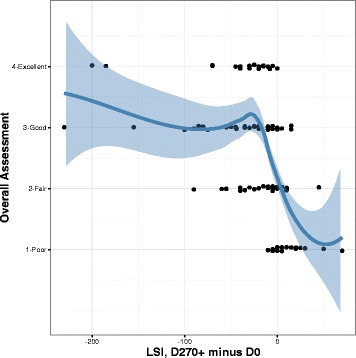
Effectiveness of regionally-specific immunotherapy in dogs versus change in lesion severity. The change in LSI equals the LSI at D270+ minus the LSI at D0. The line is a locally weighted scatterplot smoother with the shaded area showing the 95% confidence interval around the point-wise mean

The response classification at D270+ significantly correlated with a reduction in pruritus severity (Fig. 3, *r* = 0.72, *p* < 0.001), a reduction in lesion severity (Fig. 4, *r* = 0.54, *p* < 0.001), the duration of therapy to D270+ (*r* = 0.24, *p* = 0.02) and the total duration of therapy as of the date of the data retrieval (*r* = 0.40, *p* < 0.001).

No adverse reactions to RESPIT were reported in the 103 evaluable dogs meeting the inclusion criteria. Seven of 286 dogs initially screened (2.4%) were suspected by pet owners to have experienced adverse reactions to RESPIT, including three with increased pruritus, and one each with vomiting, blepharitis, restlessness, or urticaria. In 5/7 dogs the dose was decreased and RESPIT was continued. In one dog with increased pruritus during the induction phase, RESPIT was temporarily discontinued then resumed 1 month later following the induction schedule up to a lower maintenance dose. In the dog that reportedly developed hives after two injections, RESPIT was discontinued by the owner and the dog was lost to follow up.

## Discussion

In this study, 59/103 dogs (57%) had good or excellent responses to RESPIT. Similar rates have been reported in studies evaluating AIT effectiveness [5, 15–17, 19, 20]. Therapeutic extracts are likely to be imperfectly matched with dogs’ actual sensitivity with both AIT and RESPIT, perhaps accounting for the similarity in response rates.

The efficacy of immunotherapy using uniform allergen mixtures has been evaluated in two randomized controlled trials in atopic dogs, both reported only in abstract form with limited details or analysis [10, 11]. In a 12-month study of 78 dogs, Garfield found a 76% good to excellent response (>51% resolution of pruritus) to a uniform mixture of 32 aqueous allergens [10]. This was not significantly different from the response of those dogs that received either of two doses of AIT based on IDT findings. In contrast are the findings of an 8-month trial of 30 dogs in which a uniform mixture of four alum-precipitated allergens (house dust, dog dander, human dander, and grass mix) was compared to AIT. The median improvement in clinical scores (pruritus and lesion severity) was 70% in the AIT group and 18% in the group that received the uniform mixture of allergens [11]. The discrepancy in response rates between the latter study versus those of Garfield and the present study may reflect differences in the number or type of allergens in the uniform mixtures and their formulations (alum-precipitated vs. aqueous).

Beneficial effects of immunotherapy with imperfectly matched or unrelated allergens have also been reported in cats and humans [12, 21, 22]. In a feline asthma model, eosinophilic airway inflammation responded to AIT with allergens matched to experimental sensitization, but also to immunotherapy with imperfectly matched or unrelated allergens [21]. Cats dually sensitized to both Bermuda grass allergen and house dust mite given AIT to either allergen displayed decreased eosinophilic airway inflammation and higher levels of CD4+ CD25+ FoxP3+ Treg cells compared to placebo-treated cats. Differences were found in the immunological responses of cats given sensitivity-matched allergens versus those given unrelated allergens. Cats monosenesitized to Bermuda grass allergen displayed evidence of lymphocyte hypoproliferation during immunotherapy with Bermuda grass allergen, whereas Bermuda grass sensitized cats displayed lymphocyte hyperproliferation with house dust mite immunotherapy. The authors concluded that sensitizing allergens and those used in AIT need not be identically matched in order to provide a clinical benefit. Analogous findings have been reported in humans sensitive to both birch and grass pollen [22]. Sublingual immunotherapy with either birch or grass pollen led to clinical improvement and lower nasal eosinophil counts during both pollen seasons, although the improvement was greater when both were given.

Whereas perfectly matching an atopic dog’s clinical sensitivity is the objective of AIT, the mechanism of action of RESPIT may be both allergen-specific and non-specific. Phylogenetically related allergens frequently cross react on IDT in atopic dogs [23]. About 30 major groups of cross-reactive botanical proteins have been identified [24]. The RESPIT extract used in this study contained 20 allergens representing a spectrum of botanically related allergen groups and house dust mites. RESPIT may imperfectly match an atopic dog’s actual sensitivities, but include some allergen-specific epitopes as well as panallergens (e.g. profilins, polcalcins, and non-specific lipid transfer proteins) common to distinct allergen groups. Although panallergens are widely distributed in nature with highly conserved amino acid sequence regions, structures, and functions, their clinical significance in human allergy is unclear [24, 25].

In the present study, the median duration of therapy at the time of the D270+ evaluation was 12 months and the majority of D270+ evaluations (74/103) occurred between 9 and 15 months after beginning RESPIT (Fig. 2). The slight correlation between the days until D270+ and response classification, and the moderate correlation between the total length of therapy and response classification at D270+ likely reflect pet owners’ higher level of compliance when satisfied with their dogs’ response. The calendar month during which D270+ evaluations took place did not correlate with the response to RESPIT (Fig. 1). Taken together, these findings suggest that the possible confounding variable of seasonality did not account for the clinical improvement detected in this study.

Similar to a number of retrospective studies of AIT in atopic dogs [5], these results suggest that a dog’s response to RESPIT cannot be predicted from their age, weight, gender, or from the seasonality of their signs. The relatively low number of dogs of any given breed did not allow for rigorous analysis of the possible correlation of breed and response classification. Neither D0 pruritus severity nor lesion severity significantly correlated with the response classification. The dataset did not allow for precise reporting of the duration of clinical signs prior to RESPIT therapy.

For many pet owners, pruritus is the most important burden of canine atopic dermatitis [26]. Pruritus severity was scored with the validated visual analog scale [10], however, lesion severity was scored with an ad hoc scale (LSI). The third iteration of the canine atopic dermatitis extent and severity index, the only validated lesion severity scale available when the data collection began, was not practical for routine use in a clinical setting [27].

Immunotherapy with irrelevant allergens could, in theory, lead to the development of clinical sensitivity. This outcome may occur with either RESPIT or AIT with imperfectly matched allergens. However, this study found that the prevalence of adverse reactions to RESPIT (2.4%) was at the low end of the wide range reported for AIT (5–50%) [4, 5]. In a small study, immunotherapy with irrelevant allergens did not lead to the development of clinical signs of atopic dermatitis in normal dogs [28].

In children with rhinitis or asthma who are sensitive to house dust mite, AIT may have a tolerogenic effect, preventing the sensitization to additional allergens by inducing a shift from a T_H_2 to a T_H_1 allergen response [29]. Irrelevant allergens that are prescribed in either AIT or RESPIT may also confer some degree of non-specific allergen tolerance. This could explain, in part, why the reported success rates of AIT are similar when utilizing a variety of allergy testing techniques and assays that exhibit poor agreement with one another [9].

A limitation of this study was the open, retrospective design, similar to most studies on the effectiveness of AIT [5]. A placebo-controlled trial could provide a higher level of evidence concerning the efficacy of immunotherapy, but may suffer from a high rate of non-compliance during a long-term study. Forty-three percent of dogs were lost to follow up by 12 months in the prospective study of AIT by Willemse [16]. A second limitation of our study was that only 36% of the dogs initially identified met the inclusion criteria, which required a follow-up examination after 270 days of therapy. In the current study, dogs that continued to receive RESPIT prescribed by their primary care veterinarian but did not return for the D270+ examination were excluded. Allowing telephone interviews for follow-up, as have some retrospective studies of AIT [15, 20], may have resulted in excluding fewer dogs, but would not have allowed for consistent scoring of pruritus and lesion severity.

## Conclusion

This retrospective study suggests that subcutaneous RESPIT is a safe and effective alternative to AIT in atopic dogs with the advantage of avoiding the subjectivity involved in allergy testing and AIT formulation.

## Abbreviations

AD: Atopic dermatitis
AIT: Allergen immunotherapy
D0: Day 0
D270+: Days from the initial examination to the first examination to occur at least 270 days later
IDT: Intradermal test
LSI: Canine lesion severity index (an ad hoc 0–1000 scale equal to the product of lesion severity graded from 0 to 10 and the estimated percent body area affected)
PVAS: Pruritus visual analogue scale
RESPIT: Regionally-specific immunotherapy
SIA: Serum allergen-specific IgE assay
